# Strain Engineering on the Electronic and Optical Properties of WSSe Bilayer

**DOI:** 10.1186/s11671-020-03330-z

**Published:** 2020-05-04

**Authors:** Jian Guo, Congming Ke, Yaping Wu, Junyong Kang

**Affiliations:** Department of Physics, OSED, Fujian Provincial Key Laboratory of Semiconductor Materials and Applications, Jiujiang Research Insititute, Xiamen University, Xiamen, 361005 People’s Republic of China

**Keywords:** WSSe bilayer, Biaxial strain, Optical anisotropy, The first-principle calculations

## Abstract

Controllable optical properties are important for optoelectronic applications. Based on the unique properties and potential applications of two-dimensional Janus WSSe, we systematically investigate the strain-modulated electronic and optical properties of WSSe bilayer through the first-principle calculations. The preferred stacking configurations and chalcogen orders are determined by the binding energies. The bandgap of all the stable structures are found sensitive to the external stress and could be tailored from semiconductor to metallicity under appropriate compressive strains. Atomic orbital projected energy bands reveal a positive correlation between the degeneracy and the structural symmetry, which explains the bandgap evolutions. Dipole transition preference is tuned by the biaxial strain. A controllable transformation between anisotropic and isotropic optical properties is achieved under an around − 6%~− 4% critical strain. The strain controllable electronic and optical properties of the WSSe bilayer may open up an important path for exploring next-generation optoelectronic applications.

## Introduction

Two-dimensional (2D) materials with their novel properties have been showing great application prospect in next-generation electronic devices. As a promising candidate, 2D-layered transition metal dichalcogenides (TMDCs) with tunable bandgap were widely studied over the past decade and were intensively exploited as tunneling field-effect transistors [1], light-emitting diodes, photodetectors [2, 3], sensors [4], and so on.

Beyond the highly symmetrical MX_2_ (*M* = Mo, W; *X* = S, Se, Te) configuration, new Janus structural TMDCs, with the chemical formula of MXY (*M* = Mo, W; *X* ≠ *Y* = S, Se, Te) have attracted increasing interest due to their distinctive optical and electronic properties. The monolayer MXY is constructed by two different chalcogen atom layers marked as A, A’ and one transition-metal atom layer B to form an ABA’ atomic stacking. Compared with that of MX_2_, MXY possesses an asymmetry-ordered configuration with the breaking of mirror symmetry, which leads to a vertical dipole and enhanced Rashba spin-orbit coupling [5]. Geometric and electronic structures of Janus WSSe have already been reported and proved to have plenty of distinguishing features different from both WS_2_ and WSe_2_. For instance, the hydrogen evolution reaction catalytic activity of WSSe was found superior to that of current TMD-based catalysts [6]. The WSSe field-effect transistors also have achieved better performance in electron mobilities and *I*_ON_/*I*_OFF_ ratio than that of conventional TMD monolayers [7]. Despite the exciting characters of the intrinsic monolayer, Janus TMDCs with bilayer and multilayer thickness and various stacking structures may possess profound physical connotations considering the asymmetry of the MXY configuration. For example, the Se-S-Se-S-ordered WSSe bilayer was predicted to improve the efficiency of photoelectric conversion efficiency for solar cell applications [8].

Based on the unique Janus TMDC materials, realizing an accurate control of their electronic and optical properties is vital to meeting the multiple needs of device design. Electric field [9, 10], strain [11, 12], surface decoration [13, 14], and magnetic doping [15–17] have been proven as effective means to modulate the electronic and optical behaviors of 2D TMDCs. Among these methods, strain engineering is reversible with the controllable process, while without generating additional lattice defects and damage in the materials. Besides, strain engineering will alter the structural symmetry, which may give rise to the polarized characteristics of 2D materials and endow them with great prospects in future applications. As has been reported, the strained WSe_2_ monolayers show obvious variation in electronic band structure [18–22] and demonstrate unique advantages in the applications of photoactive devices [23], valleytronics [18, 24], photodetectors [25], and anode material for Li-ion battery [26]. Nevertheless, strain engineering on the electronic and optical properties, such as band evolution and optical anisotropy of 2D Janus WSSe bilayer has not yet been reported so far.

In this work, we perform an investigation on the strain modulation of the electronic and optical properties of WSSe bilayer through the first-principle density function calculations. The investigation is initiated with the determination of the most favorable stacking order of the bilayer. Strain-dependent band structures of the three stable configurations are calculated. The bandgaps of WSSe bilayers are tailored and the atomic orbital contribution is revealed to understand the related mechanism. Optical anisotropy is also modulated by tuning the dielectric properties through the applied strain. A controllable transformation between anisotropic and isotropic optical properties is demonstrated.

## Computational Method

All theoretical calculations are based on the density functional theory (DFT) with the generalized gradient approximation (GGA). The accurate projector-augmented wave (PAW) method, as implemented in the Vienna *Ab-initio* Simulation Package (VASP) [27–29] code is used. A slab model with a 1 × 1 unit cell is constructed, and a 20 Å vacuum layer along the *z* direction is used to minimize artificial interactions between neighboring slabs. The valence electron configurations of W, S, and Se atoms adopted are *5p*^*6*^*5d*^*4*^*6s*^*2*^, *2s*^*2*^*3p*^*4*^, and 4*s*^2^4*p*^4^, respectively. The GGA [30] with Perdew-Burke-Ernzerhof (PBE) [31] parameterization is employed as the exchange-correlation functional. Electron wave functions are expanded in plane waves with an energy cutoff of 400 eV. The Brillouin zone is sampled with a 19 × 19 × 1 Monkhorst-Pack grid of *k* points. The DFT-D2 dispersion correction method is included in the structural relaxation and electronic structure calculations to correctly describe the effect of van der Waals integrations. All atomic degrees of freedom, including lattice constants, are fully relaxed with self-consistent convergence criteria of 0.01 eV/Å and 10^-6^ eV for the atomic forces and total energy, respectively.

## Results and Discussion

**Fig. 1:**
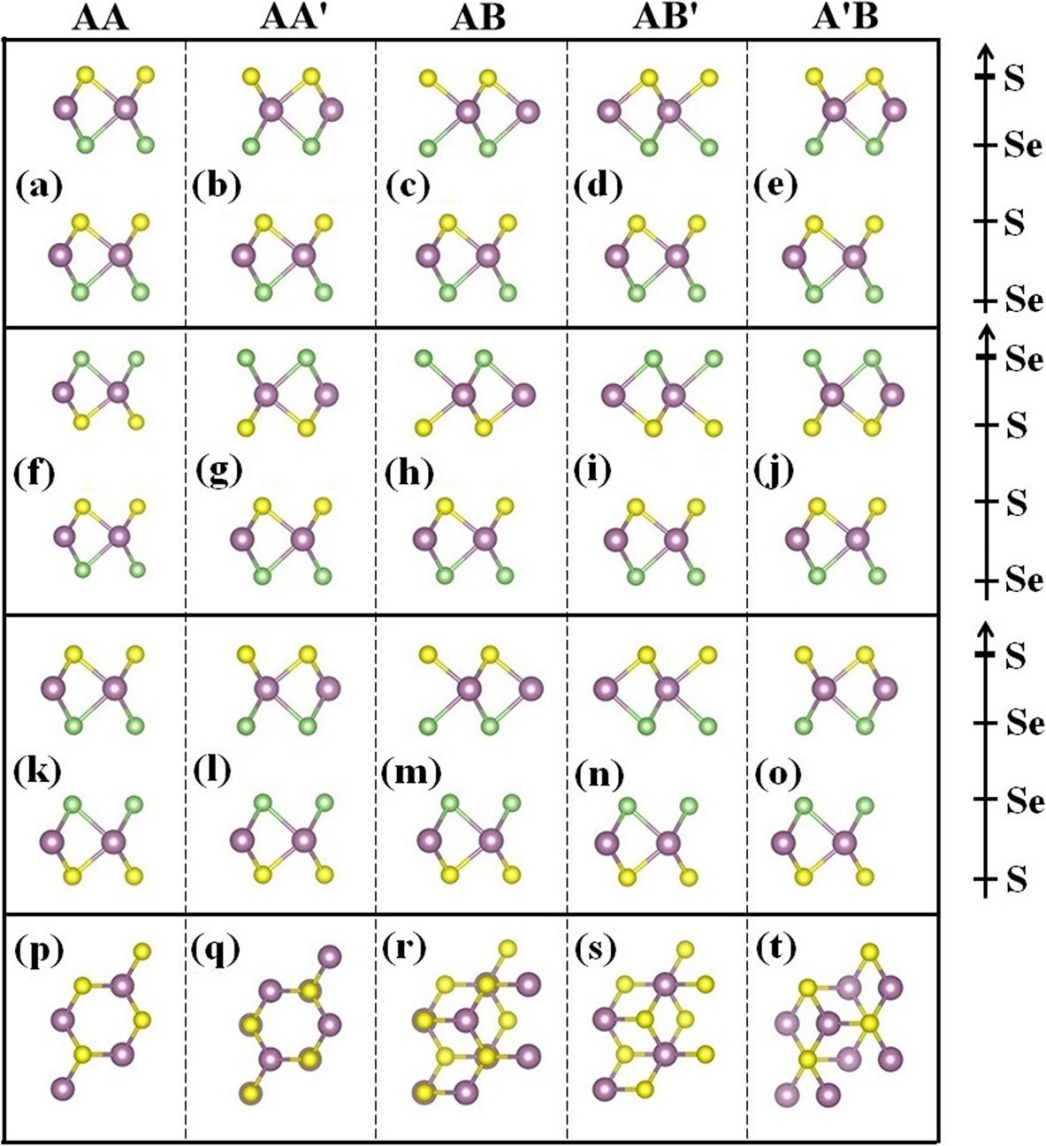
Top and side views of the atomic configuration of WSSe bilayer. The purple balls represent the W atoms, and the yellow and green balls represent the S and Se atoms, respectively

The Janus WSSe monolayer has a hexagonal lattice, where the unit cell consists of a middle W atom in its planar honeycomb lattice that bonded three-coordinately with the surface S and Se atoms. The optimized lattice constant of WSSe is 3.23 Å with the W-S and W-Se bond lengths of 2.42 and 2.53 Å, respectively, which are aligned with the previous reported values [32]. According to the structural symmetry, five different stacking configurations of WSSe bilayer are taken into account, which is marked as AA, AA’, AB, AB’, and A’B, respectively. For each stacking, three different orders of chalcogen layers: S-Se-S-Se, Se-S-S-Se, and S-Se-Se-S are considered. All equilibrium geometric configurations of the WSSe bilayer are depicted in Fig. 1. Each configuration is fully relaxed respectively to optimize the interlayer spacing.

In order to determine the structural stability of the WSSe bilayer quantitatively, the binding energies *E*_b_ of all above geometric configurations are calculated from the relation:

$$ {E}_{\mathrm{b}}=2{E}_{\mathrm{WSSe}}-{E}_{\mathrm{b}\mathrm{ilayer},} $$

where *E*_bilayer_ and *E*_WSSe_ are the total energies of WSSe bilayer and monolayer, respectively.

**Fig. 2:**
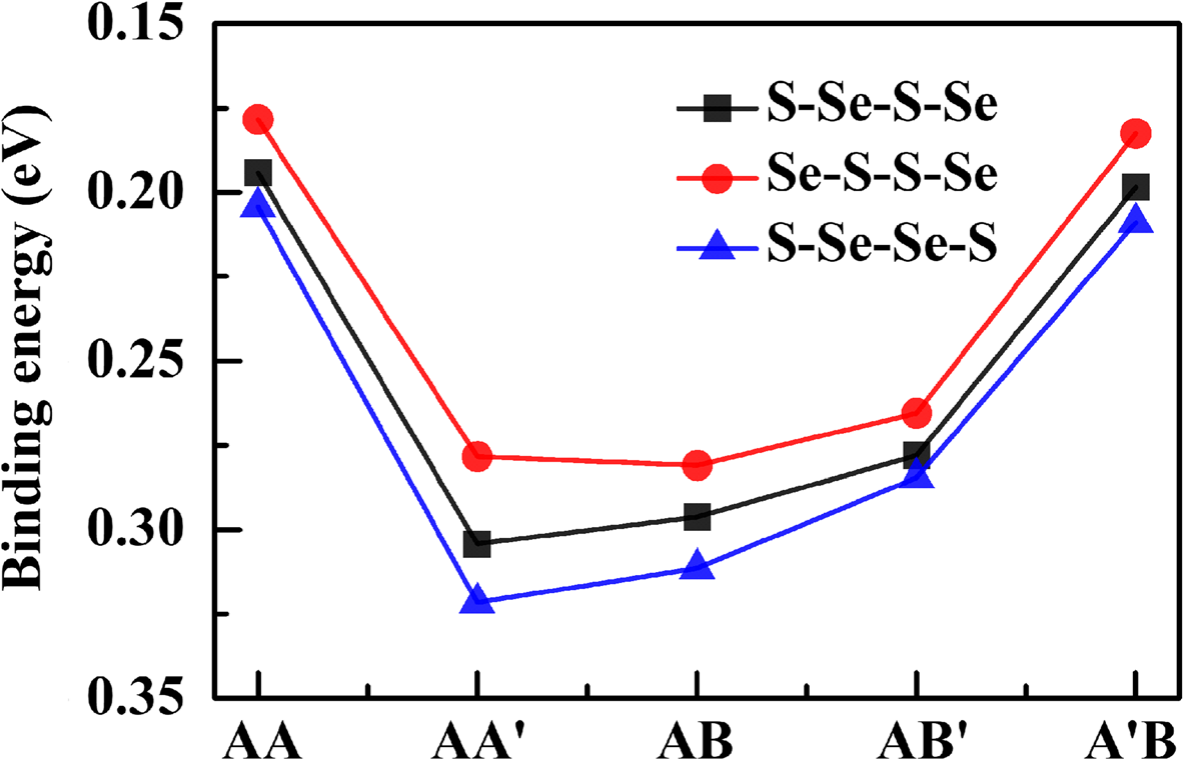
Binding energies of all the equilibrium geometric configurations of WSSe bilayer

As shown in Fig. 2, for all the stacking structures, chalcogen layers with the order of S-Se-Se-S possess the largest binding energy, while the reversed order Se-S-S-Se has the smallest binding energy. In addition, it is visualized that AA’, AA’, and AB are the most stable stacking configurations of S-Se-Se-S, S-Se-S-Se, and Se-S-S-Se orders, with the binding energies of 0.322, 0.304, and 0.281 eV, respectively. This indicates that the Janus WSSe bilayer prefers to form a bilaterally symmetrical AA’ stacking with the S-Se-Se-S chalcogen order, which is different from the MoSSe/WSSe heterostructure of AB stacking [33].

Considering the most stable stacking structures mentioned above for each chalcogen order, both the electronic and optical properties are profoundly investigated. For convenience, the AA’ stacking with S-Se-S-Se structure, the AB stacking with Se-S-S-Se structure, and the AA’ stacking with S-Se-Se-S structure are named as *I*_1_, *I*_2_, and *I*_3_, respectively, in the following discussion.

**Fig. 3:**
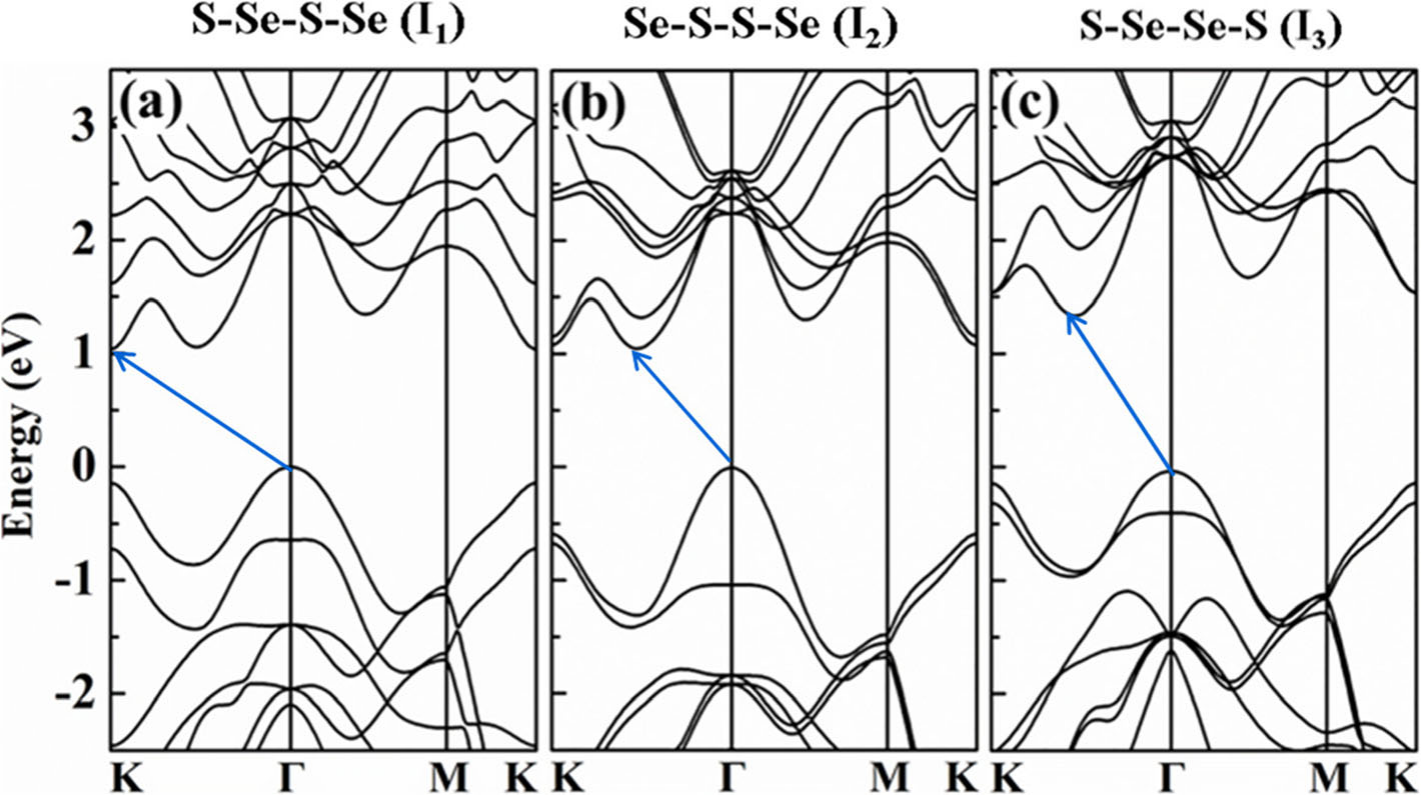
Band structures of *I*_1_, *I*_2_, and *I*_3_, respectively, where the bandgaps are denoted by the blue arrows

Band structures of the Janus WSSe bilayers *I*_1_, *I*_2,_ and *I*_3_ are calculated, as shown in Fig. 3. All the three configurations exhibit a fundamental indirect bandgap structure, which is similar to that of the pure bilayer WS_2_ and WSe_2_. The valence band maximums (VBM) are all locating at *Γ* point, while the conduction band minimum (CBM) locating at *K* point for *I*_1_, and situating between *K* and *Γ* points for both *I*_2_ and *I*_3_. The indirect bandgap of *I*_3_ is calculated to be roughly 1.3 eV, slightly larger than that of *I*_1_ and *I*_2_ whose bandgaps are approximately 1.0 eV. Despite that the bandgaps are underestimated without the screened hybrid HSE06 functional, the band structure distributions have no significant change, and thus, the underestimation will not substantially influence the evolution tendency of the electronic properties under the strain modulation.

**Fig. 4:**
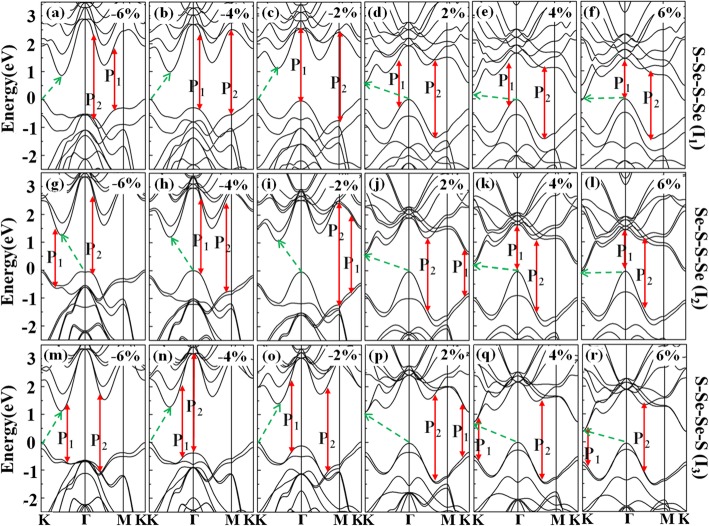
**a**–**r** Band structures of *I*_1_, *I*_2_, and *I*_3_ with different strains of − 6%, − 4%, − 2%, 2%, 4%, and 6%, respectively. The bandgaps are denoted with the dashed green arrows, while red solid arrows depict the principal interband transitions of *P*_1_ and *P*_2_, respectively

Strain engineering is a promising method for manipulating the structural symmetry and the interlayer interaction, which could give rise to plenteous charming phenomena. To study the electronic structures of WSSe bilayers modulated by the applied strain, the energy bands are analyzed, as illustrated in Fig. 4a–r. When a compressive strain ranging from − 6 to − 2% is applied, the original VBM at *Γ* point changed to *K* point for *I*_1_ and *I*_3_ configurations, while shows little variety for *I*_2_. The original CBM at *K* point shifts to the position between *Γ* and *K* points for all the three structures. Once the tensile strain in the region of 2%~6% is employed, the VBM remains at *Γ* point while the CBM is all locating at the K point.

**Fig. 5:**
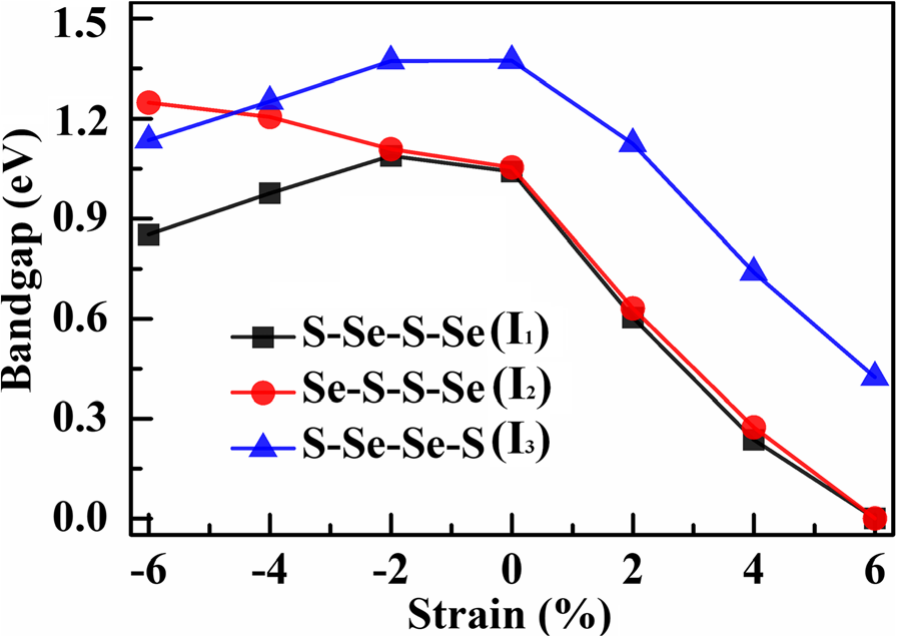
The bandgap (*E*_g_) versus the applied strains for the *I*_1_, *I*_2_, and *I*_3_ structures

Figure 5 summarizes the strain-dependent bandgap for the three structures. It is apparent at a glance that the responses of the bandgap to compressive strain and tensile strain are not only with inequal responsivity but also with different gradients as the applied strain increases. The bandgap is less sensitive to compressive strain, while dramatically decreases with the enhanced tensile strains. As the compressive strain increases, the CBM of both *I*_1_ and *I*_3_ is uplifted to higher energy, whereas that of *I*_2_ is downshifted to lower energy, resulting in a slight decrease for *I*_2_ and increase for *I*_1_ and *I*_3_ in the indirect bandgaps. In the presence of the tensile strain, the CBM enormously decreases while the VBM rises gently. The indirect bandgap thus exhibits a conspicuous diminishment and decreases sharply when the tensile strain reaches 6%. Compared with that of the strained Janus WSSe monolayer [34], the bandgaps of I_1_ and I_3_ show generally similar evolution with both compressive and tensile strain modulations, while the bandgap of I_2_ behaves oppositely under the compressive strains.

**Fig. 6:**
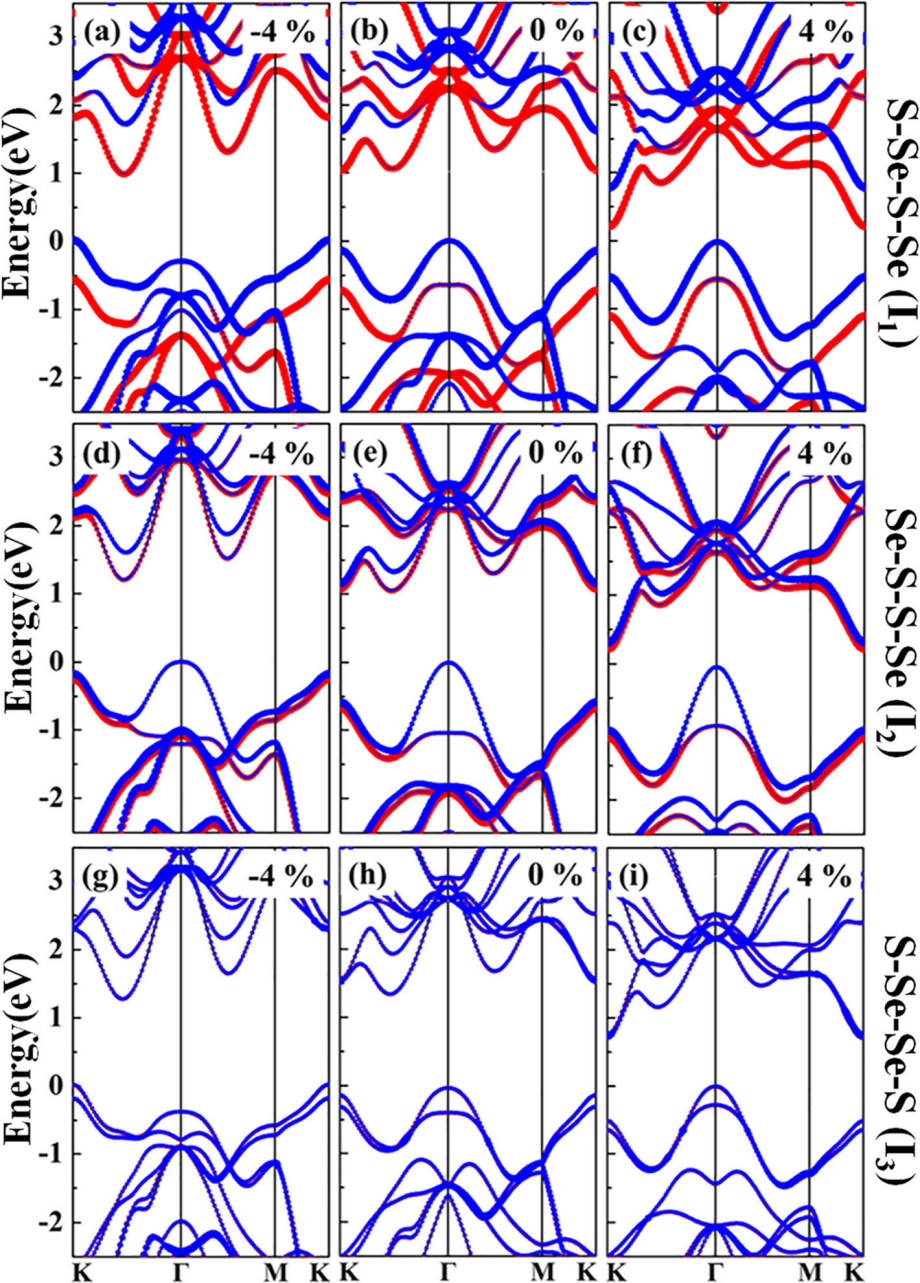
Atomic orbital projected energy bands of *I*_1_, *I*_2_, and *I*_3_ structures under the strains of − 4%, 0, and 4%, respectively. Blue and red colors mean orbital contributions from the upper and the lower layers, respectively

In order to gain an insight into the electronic structure of WSSe bilayer in the presence of the strains, the atomic orbital projected energy band is studied, as seen in Fig. 6. Owing to its center inversion symmetry (Fig. 1l), the orbitals of the upper and lower layers for *I*_3_ are energy degenerate, which make identical contributions to the band structure. On the contrary, because of the structure inversion asymmetry of *I*_1_ and *I*_2_, the orbitals of the upper and lower layers are splitted. The above results suggest that there is a positive correlation between the degeneracy and the structural symmetry. Owing to the center inversion symmetry of *I*_3_ stacking, the orbitals of the upper and lower layers for *I*_3_ are energy degenerate, which make identical contributions to the band structure regardless of the varying strains. As depicted in Fig. 6g–i, both the CBM and VBM equally derived from the two WSSe layers. On the contrary, because of the structural inversion asymmetry of I_1_ and I_2_, the orbitals of the two layers are splitted, as shown in Fig. 6a–c and Fig. 6d–f. The original *I*_1_ structure exhibits a typical type-II heterostructure, with the CBM and VBM contributed from the lower and upper WSSe Janus layers, respectively. The band alignment does not vary under either the compressive or tensile strains (Fig. 6a–c). As for the *I*_2_ stacking without and with a compressive strain, the CBM comes from both the two layers, and the VBM originates from the upper layer (Fig. 6d, e). The *I*_2_ heterostructure changes to a type-II band alignment under the tensile strain (Fig. 6f), which indicates a promising prospect for developing high-performance optoelectric conversion and energy storage devices [35].

**Fig. 7:**
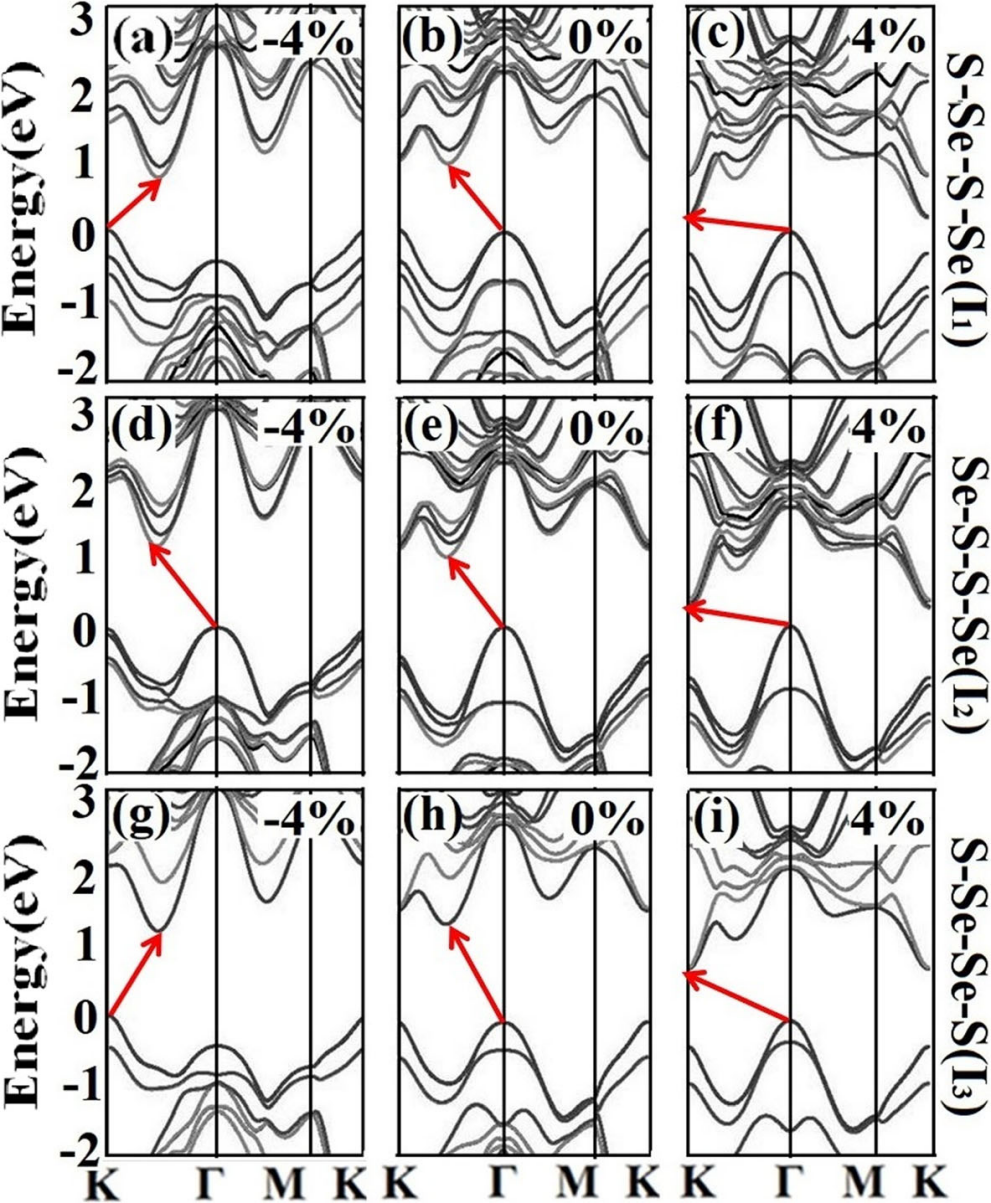
**a**–**i** Band structures of *I*_1_, *I*_2_, and *I*_3_ under the strains of − 4%, 0, and 4% with the consideration of SOC effect, where the black and blown colors mean the up and down spins, respectively. The bandgaps are denoted by the red arrows

To further explore the spin-orbit coupling (SOC) effect in the strain engineering in the WSSe bilayer, the band structures with the consideration of SOC are further calculated without and with the strains of − 4% and 4%, as shown in Fig. 7. It is found that, for all the three configurations, the band structures including the momentum positions of VBM and CBM, the bandgaps, and band distributions show similar evolution tendency with the varying strains. This suggests that the strain modulation regularity still remains, and the SOC effect does not obviously influence the main conclusions.

**Fig. 8:**
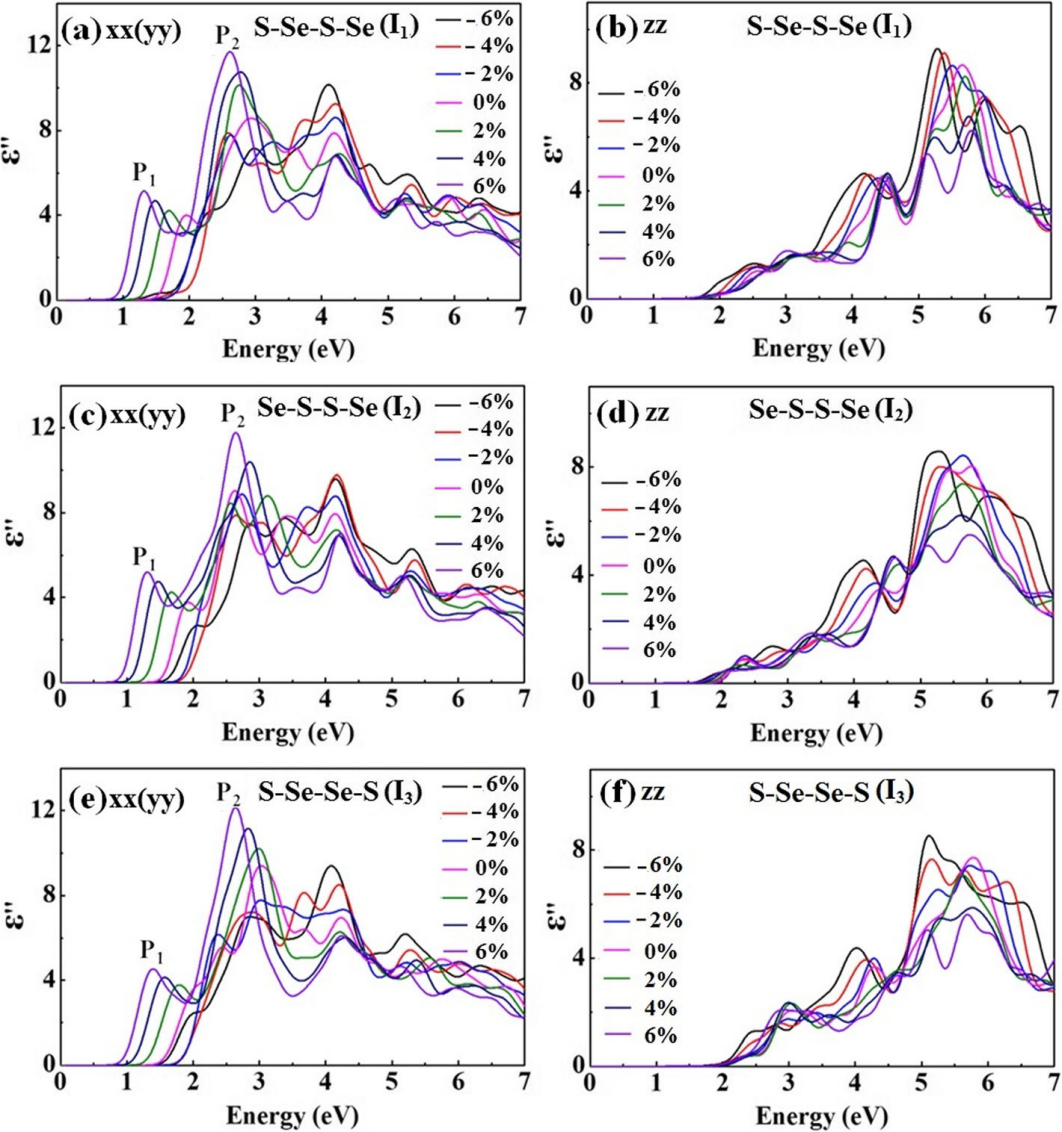
The imaginary parts of calculated optical dielectric function ε^”^_xx_ (ε^”^_yy_) and ε^”^_zz_ for *I*_1_ (**a**, **b**), *I*_2_ (**c**, **d**), and *I*_3_ (**e**, **f**) WSSe bilayer versus the applied strain, respectively

With the aim to modulate the optical properties of the WSSe bilayer, the response of the dielectric function under external varying strain is studied. Figure 8 displays the complex dielectric function ε^”^_xx_ (ε^”^_yy_) and ε^”^_zz_ of WSSe bilayer versus the applied strain. ε^”^_xx_ (ε^”^_yy_) is found to shift to lower energies with the increasing tensile strain, and on the contrary, shift to the higher energy region while a compressive strain is applied. Compared with the unstrained WSSe bilayer with the dipole transition of 0.79, 1.18, and 1.15 eV, respectively for *I*_1_, *I*_2_, and *I*_3_ structures, the strain modulation is able to obtain a wide-range transition energy from 0.24 to 1.47 eV in near-infrared and mid-infrared area, which could be offering extensive possibilities for assorted detectors, for instance, infrared detector and pyroelectric detector.

The main peaks in the imaginary part of the dielectric function labeled as *P*_1_ and *P*_2_ in Fig. 8a, c, and e could be assigned to the principal interband transitions. This is achieved by fitting the peak energies in Fig. 8 with that of the interband transitions in Fig. 4. When a strain ranging from − 6 to 6% is applied, the peak energies of *P*_1_ and *P*_2_ increase firstly and then decrease. Regardless of the strains, both the *P*_1_ and *P*_2_ peaks are found to take place in the energy range of 1.3–3.0 eV, which exhibit great enhanced response in a wide spectrum from ultraviolet, visible to the near-infrared area. The widely distributed peaks should be suitable for the design of multiband metamaterial emitters with promising photoelectric applications.

Controllable anisotropy of WSSe bilayer through the strain engineering is further investigated. Compared with that of ε^”^_xx_ (ε^”^_yy_), ε^”^_zz_ exhibits insignificant variation regardless of the tensile or compressive strain. This manifests the fact that the imaginary part of the dielectric function possesses different response properties with the increased strain. Without the strain, the ε^”^_xx_ (ε^”^_yy_) and ε^”^_zz_ are anisotropy with E||ĉ transformation preference for all the *I*_1_, *I*_2_, and *I*_3_ structures. For either *I*_1_ or *I*_3_, while a compressive strain is applied, the anisotropy of dipole transition is firstly enhanced and then weakened and that with the tensile strain is always enhanced. Nevertheless, the anisotropy of *I*_2_ is enhanced with the increasing tensile strain and becomes weakened once a compressive strain is introduced. An isotropy of dipole transition occurs when the compressive strain continues to increase to − 6%~− 4%, where both E||ĉ and E⊥ĉ possess equal transformation preference. Thus, the WSSe bilayer with a suitable strain modulation will be leading to a transition from optical anisotropy to isotropy. Since the excitonic effect usually plays an important role in the optical absorption [36, 37], the dielectric function determined dipole transition preference may be explored for the potential optoelectronic applications with an electroluminescence process.

As has been demonstrated that some typical TMDC monolayers with 2H phase have the same hexagonal lattices and similar characters in their monolayer band structures [5, 33, 38, 39]. Therefore, the Janus monolayer and bilayer derived from these TMDC materials, such as MXY (*M* = Mo or W, *X*/*Y* = S, Se, or Te, and *X* ≠ *Y*), would be expected to possess similar band structures [8, 32] and thus the similar electronic and optical properties, as well as evolution tendency with strain modulation. Therefore, the main calculation results will have certain universality in 2H-TMDC Janus materials. Going through the previous reports, the mechanical properties of out-of-plane bended MoS_2_ thin films have been revealed [40], the electronic and optical properties of TMDC compounds have been studied [22], and the energy gaps of monolayer and Janus heterobilayer TMDCs have been demonstrated to control the electric field [41]. Comparing with these works, we provide a series of innovative results in strain-modulated electronic and optical properties of 2D Janus WSSe bilayer, which enriches the physical connotation of the Janus materials and provides a promising control strategy towards the application of next-generation electronic and optoelectronic nanodevices.

## Conclusion

In summary, the strain dependence of electronic and optical properties of the WSSe bilayer is systematically studied. By comparing the binding energies of different stackings, the most favorable configuration of the WSSe bilayer is determined. The WSSe bilayer preserves an indirect bandgap structure, which is sensitive to the external stress. The bandgap of all the stable structures can be tailored from the semiconductor to metallicity under to obtain a wide-range spectrum in near-infrared and mid-infrared area. Atomic orbital projected energy bands reveal a positive correlation between the degeneracy and the structural symmetry, which explains the bandgap evolutions. Dipole transition preference is investigated from the dielectric properties and tuned by the biaxial strain. Under around − 6%~− 4% critical strain, a controllable transformation between anisotropic and isotropic optical properties is realized. The strain-modulated electronic and optical behaviors of Janus WSSe bilayer possess a wide application prospect in next-generation electronic and optoelectronic nanodevices.

## Data Availability

All data generated or analyzed during this study are included in this published article.

## Abbreviations

2D: Two-dimensional
CBM: Conduction band minimum
DFT: Density functional theory
SOC: Spin-orbit coupling
TMDCs: Transition metal dichalcogenides
VBM: Valance band maximum

## References

[1] Sarkar D, Xie X, Liu W, Cao W, Kang J, Gong Y, Kraemer S, Ajayan P, Banerjee K (2015). A subthermionic tunnel field-effect transistor with an atomically thin channel. Nature..

[2] Withers F, Del Pozo-Zamudio O, Mishchenko A, Rooney A, Gholinia A, Watanabe K, Taniguchi T, Haigh S, Geim A, Tartakovskii A, Novoselov K (2015). Light-emitting diodes by band-structure engineering in van der Waals heterostructures. Nat Mater.

[3] Plechinger G, Korn T, Lupton J (2017). Valley-polarized exciton dynamics in exfoliated monolayer WSe_2_. J Phys Chem C.

[4] He P, Brent J, Ding H, Yang J, Lewis D, O’Brien P, Derby B (2018). Fully printed high performance humidity sensors based on two-dimensional materials. Nanoscale..

[5] Cheng Y, Zhu Z, Tahir M, Schwingenschlögl U (2013). Spin-orbit-induced spin splittings in polar transition metal dichalcogenide monolayers. EPL..

[6] Er D, Ye H, Frey N, Kumar H, Lou J, Shenoy V (2018). Prediction of enhanced catalytic activity for hydrogen evolution reaction in Janus transition metal dichalcogenides. Nano Lett.

[7] Karande S, Kaushik N, Narang D, Late D, Lodha S (2016). Thickness tunable transport in alloyed WSSe field effect transistors. Appl Phys Lett.

[8] Zhou W, Chen J, Yang Z, Liu J, Ouyang F (2019). Geometry and electronic structure of monolayer, bilayer, and multilayer Janus WSSe. Phys Rev B.

[9] Zhang F, Zhang H, Krylyuk S, Milligan C, Zhu Y, Zemlyanov D, Bendersky L, Burton B, Appenzeller A (2019). Electric-field induced structural transition in vertical MoTe_2–_ and Mo_1–x_WxTe_2–_ based resistive memories. Nat Mater.

[10] Ke C, Wu Y, Guo G, Lin W, Wu Z, Zhou C, Kang J (2018). Tuning the electronic, optical, and magnetic properties of monolayer GaSe with a vertical electric field. Phys Rev Appl.

[11] Kansara S, Gupta K, Sonvane K (2018). Effect of strain engineering on 2D dichalcogenides transition metal A DFT study. Comput Mater Sci.

[12] Li X, Zhang S, Wang Q (2017). Topological insulating states in 2D transition metal dichalcogenides induced by defects and strain. Nanoscale..

[13] Jiang J, Ni Z (2019). Defect engineering in two-dimensional materials. J Semicond.

[14] Rafiq M (2018). Carrier transport mechanisms in semiconductor nanostructures and devices. J Semicond.

[15] Liu B, Wu L, Zhao Y, Wang L, Cai M (2016). A first-principle study of magnetic variation via doping vacancy in monolayer VS_2_. J Magn Magn Mater.

[16] Liu J, Hou CC, Fu H, Sun J, Meng S (2017). Intrinsic valley polarization of magnetic VSe_2_ monolayers. J Phys Condens Matter.

[17] Luo N, Si C, Duan W (2017). Structural and electronic phase transitions in ferromagnetic monolayer VS_2_ induced by charge doping. Phys Rev B.

[18] Peng G, Lo P, Li W, Huang Y, Chen Y, Lee C, Yang C, Cheng S (2019). Distinctive signatures of the spin- and momentum-forbidden dark exciton states in the photoluminescence of strained WSe_2_ monolayers under thermalization. Nano Lett.

[19] Gujarathi D, Solanki G, Deshpande M, Agarwal M (2005). Band gap in tungsten sulphoselenide single crystals determined by the optical absorption method. Mater Sci Semicond Process.

[20] Ni Z, Yu T, Lu Y, Wang Y, Feng Y, Shen Z (2008). Uniaxial strain on graphene: raman spectroscopy study and band-gap opening. ACS Nano.

[21] McCann E (2006). Asymmetry gap in the electronic band structure of bilayer graphene. Phys Rev B.

[22] Rafael R, Silva-Guillén J, López-Sancho M, Guinea F, Cappelluti E, Ordejón P (2014). Electronic properties of single-layer and multilayer transition metal dichalcogenides MX_2_(M= Mo, W and X= S, Se). Ann Phys (Berlin).

[23] Li L, Carter P (2019). Defect-mediated charge-carrier trapping and nonradiative recombination in WSe_2_ monolayers. J Am Chem Soc.

[24] Yang Q, Yuan R, Guo Y (2019). Valley switch effect based on monolayer WSe_2_ modulated by circularly polarized light and valley Zeeman field. J Phys D Appl Phys.

[25] Yea L, Wang P, Luo W, Gong F, Liao L, Liu T, Tonga L, Zang J, Xu J, Hu W (2017). Highly polarization sensitive infrared photodetector based on black phosphorus-on-WSe_2_ photogate vertical heterostructure. Nano Energy.

[26] Rehman J, Roshan A, Nisar A, Lv X, Guo C (2019). Theoretical investigation of strain-engineered WSe_2_ monolayers as anode material for Li-ion batteries. J Alloys Compd.

[27] Kresse G, Hafner J (1994). Ab initio molecular-dynamics simulation of the liquid-metal-amorphous-semiconductor transition in germanium. Phys Rev B.

[28] Kresse G, Furthmüller J (1996). Efficient iterative schemes for ab initio total-energy calculations using a plane-wave basis set. Phys Rev B.

[29] Kresse G, Joubert D (1999). From ultrasoft pseudopotentials to the projector augmented-wave method. Phys Rev B.

[30] Kim Y, Hummer K, Kresse G (2009). Accurate band structures and effective masses for InP, InAs, and InSb using hybrid functionals. Phys Rev B.

[31] Perdew J, Burke K, Ernzerhof M (1996). Generalized gradient approximation made simple. Phys Rev Lett.

[32] Kandemir A, Sahin H (2018). Bilayers of Janus WSSe: monitoring the stacking type via the vibrational spectrum. Phys Chem Chem Phys.

[33] Li F, Wei W, Zhao P, Huang B, Dai Y (2017). Electronic and optical properties of pristine and vertical and lateral heterostructures of Janus MoSSe and WSSe. J Phys Chem Lett.

[34] Chaurasiya R, Dixit A, Pandey R (2018). Strain-mediated stability and electronic properties of WS_2_, Janus WSSe and WSe_2_ monolayers. Superlattice Microst.

[35] Ke C, Tang W, Zhou J, Wu Z, Li X, Zhang C, Wu Y, Yang W, Kang J (2019). Stress engineering on the electronic and spintronic properties for a GaSe/HfSe_2_ van der Waals heterostructure. Appl Phys Express.

[36] Kuklin A, Ågren H (2019). Quasiparticle electronic structure and optical spectra of single-layer and bilayer PdSe_2_: proximity and defect-induced band gap renormalization. Phys Rev B.

[37] Shi H, Pan H, Zhang Y, Yakobson B (2013). Quasiparticle band structures and optical properties of strained monolayer MoS_2_ and WS_2_. Phys Rev B.

[38] Zhang Y, Ye H, Yu Z, Liu Y, Li Y (2019). First-principle study of square phase MX_2_ and Janus MXY (M=Mo, W; X, Y=S, Se, Te) transition metal dichalcogenide monolayers under biaxial strain. Phys E.

[39] Guo S, Dong J (2018). Biaxial strain tuned electronic structures and power factor in Janus transition metal dichalchogenide monolayers. Semicond Sci Technol.

[40] Zhu Y, Wang P, Xiao S, He S, Chen J, Jiang Y, Wang Y, He J, Gao Y (2018). Manipulating three-dimensional bending to extraordinarily stiffen two-dimensional membranes by interference colors. Nanoscale..

[41] Liu Z, Lin Y, Cao C, Zou S, Xiao J, Jin X, Chen L (2018). First-principle study of electronic and sodium-ion transport properties of transition-metal dichalcogenides. Int J Mod Phys B.

